# Long-Term Outcomes of Preoperative Botulinum Toxin Use in Abdominal Wall Reconstruction: A Propensity-Matched Analysis of More Than 5000 Patients Over 5 Years

**DOI:** 10.1093/asjof/ojag055

**Published:** 2026-03-27

**Authors:** Yousef Tanas, Stephen Chen, Philong Nguyen, Joshua Wang, Haidara Bohsas, Dora Lena Fedorcsak, Tue Dinh

## Abstract

**Background:**

Preoperative botulinum toxin (BTX) has been proposed to facilitate myofascial medialization and tension reduction in abdominal wall reconstruction (AWR), but long-term comparative outcomes are unclear.

**Objectives:**

To compare short- and long-term postoperative outcomes after AWR in patients receiving preoperative BTX vs no BTX.

**Methods:**

We performed a retrospective cohort study using the TriNetX National Health Research Network. Adults undergoing AWR were identified by ICD-10/CPT codes and assigned to BTX or no-BTX cohorts. Propensity score matching (1:1) balanced demographics, comorbidities, related procedures, and medications at each follow-up point. Outcomes included hernia recurrence, infections, wound disruption, seroma, hematoma, surgical site infection (SSI), and reoperation, assessed at 6 months, 1, 3, and 5 years.

**Results:**

At 6 months, BTX was associated with lower hernia recurrence (32.1% vs 35.6%; *P* = .013) but higher risks of infections (14.4% vs 10.5%; *P* < .001), wound disruption (8.7% vs 4.7%; *P* < .001), hematoma (1.3% vs 0.5%; *P* = .003), SSI (3.9% vs 2.4%; *P* = .002), and reoperation (10.7% vs 7.8%; *P* = .001). From 1 to 5 years, recurrence rates no longer differed significantly between groups (eg, 5 years: 42.6% vs 44.8%; *P* = .112). The BTX cohort continued to demonstrate higher rates of infections, wound disruption, hematoma, SSI, and reoperation across later time points (all *P* < .05).

**Conclusions:**

Preoperative BTX for AWR was linked to an early reduction in recurrence but higher postoperative complications and reoperation over time. These observational data highlight the need for standardized BTX protocols and prospective trials to define patient selection and to balance potential short-term mechanical benefits against complication risks.

**Level of Evidence: 3 (Risk):**

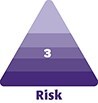

Since its introduction to clinical medicine in 1989 to treat strabismus and blepharospasm, botulinum toxin (BTX) has been used widely in the field of plastic surgery.^1^ In 2024, the American Society for Plastic Surgery reported over 9 million botulinum toxin procedures, making it the most common minimally invasive procedure of the year.^2,3^ First used in the context of aesthetics to soften glabellar frown lines, botulinum toxin now has widely expanding indications for both aesthetic and nonaesthetic procedures.^4^ Common aesthetic indications include the treatment of dynamic wrinkles, platysma bands, facial asymmetry secondary to facial nerve paralysis, and masseter reduction for facial slimming.^5,6^ Common reconstructive indications include treatment of select pain-related disorders, skeletal muscle activity disorders, exocrine gland hyperfunction, wound healing, and prosthetic breast reconstruction and augmentation.^7^

In the reconstructive setting, botulinum toxin has become an important adjunct for preoperative optimization of complex abdominal wall reconstruction (AWR).^8-10^ Abdominal wall reconstruction refers to a surgical procedure that restores abdominal wall integrity, typically performed in patients with large, recurrent, or loss of domain hernias.^11,12^ Although highly individualized, this procedure follows a general pattern of dissection, fascial closure to midline, mesh reinforcement, and occasionally flap coverage.^13,14^

Repairing large hernias, especially those with loss of domain, presents with many challenges, including high rates of infection, pain, and greater risk of recurrence.^15-17^ Furthermore, achieving durable midline fascial closure is often difficult due to retracted, shortened, or rigid abdominal wall musculature.^18^

Preoperative administration of botulinum toxin has recently been introduced to help address these concerns. Botulinum toxin is a potent neurotoxin that blocks the release of acetylcholine from nerve endings, reducing muscle spasticity.^19^ In AWR, botulinum toxin induces temporary flaccid paralysis of the lateral abdominal wall muscles, increasing abdominal domain and allowing midline fascial closure.^20,21^ Early cohort and systematic studies show that botulinum toxin has been safe and effective in facilitating midline fascial closure with low complication and recurrence rates.^9,18,22,23^ However, current literature remains limited by small sample sizes and short follow-up. The long-term durability of BTX-facilitated repairs has not yet been well defined.

The aim of this study is to evaluate the long-term outcomes of preoperative botulinum toxin use in AWR, with a focus on infections, wound disruption, seroma, hematoma, surgical site infection, reoperation, and hernia recurrence. Via this investigation, we aim to inform preoperative guidelines and enhance risk stratification for patient selection.

## METHODS

This study was conducted using the TriNetX National Health Research Network, a federated database of de-identified electronic health record data. All data available to investigators through TriNetX are de-identified in accordance with the HIPAA Privacy Rule de-identification standard (45 CFR §164.514), including expert determination as applicable. Because this study involved secondary analysis of de-identified data and no interaction with human participants, it was exempt from informed consent and institutional review board approval was not required. The study was conducted in accordance with applicable ethical standards and the principles of the Declaration of Helsinki.

### Study Design and Patient Population

This was a retrospective cohort study conducted using the TriNetX LLC National Health Research Network database. All adult patients undergoing AWR for any indication (hernia, fistula, tissue transfer), as defined by procedure codes from the International Classification of Diseases-Procedure Coding System-10th Revisions and Current Procedural Terminology (ICD10 and CPT) were included. Patients who met query criteria were divided into 2 cohorts: BTX usage in AWR (*n* = 2584) and no BTX usage in AWR (*n* = 343,589). Each patient was followed for 180-, 365-, 1095-, and 1825-days post-surgery for outcomes. Outcomes that occurred more than 20 years ago were excluded. None of the patients in either cohort were excluded since criteria was met. Data was collected across 5 years, with outcomes assessed at 6 months, 1 year, 3 years, and 5 years. The database was queried on April 16, 2025.

### Data Source

TriNetX LLC National health Research Network is a large, continuously updated, database of de-identified electronic health records from over 100 million patients across more than 70 healthcare organizations. This database aggregates structured data, such as diagnoses, procedures, medications, laboratory values, and genomics, using coding systems like the ICD10 and CPT. All data are de-identified in accordance with the Health Insurance Portability and Accountability Act (HIPAA). Institutional review board approval was not required because no identifiable information was used.

### Outcome Measures

Primary outcomes measured at every timepoint included overall infections (sepsis, skin, subcutaneous, cutaneous abscess, cellulitis, acute lymphangitis), wound disruption, seroma, hematoma, surgical site infections, reoperation, and hernia recurrence. An exploratory outcome included panniculitis.

### Statistical Analysis

Propensity score matching was performed on 10 characteristics, resulting in balanced cohorts of 2255 patients per cohort at 6 months, 2508 patients per cohort at 1 year, 2279 patients per cohort at 3 years, and 2432 patients per cohort at 5 years. In terms of demographics, patients were matched on age at the time of AWR and gender. In terms of other diagnoses, patients were matched on diabetes mellitus, atherosclerotic heart disease, overweight/obesity, thyroid disorders, and BMI. In terms of procedures, patients were matched on bariatric surgery procedures. In terms of medications, patients were matched on antiemetics and laxatives.

After 1:1 propensity-score matching of both sets of cohorts, TriNetX's “Compare Outcomes-Measures of Association” module was used to calculate absolute risk, risk difference (through *t*-test), risk ratio, and odds ratio with 95% confidence intervals. Significance was set to *P* < .05.

## RESULTS

### Technique Overview of Preoperative Botulinum Toxin

Preoperative BTA is used to induce temporary flaccid paralysis and elongation of the lateral abdominal wall musculature to facilitate medialization and tension reduction during midline closure. In published series and a large technical meta-analysis, common regimens include 200-300 units of onabotulinumtoxinA (Botox^®^) or approximately 500 units of abobotulinumtoxinA (Dysport^®^) injected bilaterally at approximately 3-5 sites per side, typically targeting the external oblique, internal oblique, and transversus abdominis muscles, most often approximately 4 weeks prior to repair for maximal effect.^21^ Muscle layers are most commonly localized using ultrasound guidance (with CT or EMG guidance described in some reports), and injections are performed intramuscularly with distribution across the lateral muscle complex.^24^

### 6 Months

Before propensity matching, there was a total of 274,823 patients: 2271 patients in the BTX usage with AWR cohort and 272,552 patients in the no BTX usage with AWR cohort.

After propensity matching, a total of 4510 patients were included in the analysis (Table 1). In the BTX usage with AWR cohort (*n* = 2255), the mean age was 55.5 (SD 16.3). There were 1306 (57.9%) females, 918 (40.7%) males, and 31 (1.4%) unknown gender. The mean follow-up time was 158 days (SD 48.4). Medical history of the BTX usage with AWR cohort revealed 23.2% with diabetes mellitus, 19.2% with atherosclerotic heart disease, 26.9% with thyroid disorders, 45.7% classified as obese. In the no BTX usage with AWR cohort (*n* = 2255), the mean age was 55.5 (SD 16.3). There were 1306 (57.9%) females, 918 (40.7%) males, and 31 (1.4%) unknown gender. The mean follow-up time was 152 days (SD 57.5). Medical history of the BTX usage with AWR cohort revealed 23.1% with diabetes mellitus, 19.2% with atherosclerotic heart disease, 18.2% with thyroid disorders, 46.2% classified as obese.

**Table 1.** Demographic and Operative Characteristics of Patients Undergoing AWR at 6 Months

	Before matching						After matching					
	BTX		No BTX				BTX		No BTX			
	2271		272,552				2255		2255			
	*n*/mean	%/SD	*n*/mean	%/SD	*P*-value	Standardized difference	*n*/mean	%/SD	*n*/mean	%/SD	*P*-value	Standardized difference
Age (years)	55.5	16.3	54.4	18.0	.003	0.066	55.5	16.3	55.5	16.3	.983	0.001
Gender												
Female	1306	57.9%	129,430	48.8%	<.001	0.183	1306	57.9%	1306	57.9%	1	<0.001
Male	918	40.7%	127,221	48.0%	<.001	0.147	918	40.7%	918	40.7%	1	<0.001
Unknown	31	1.4%	8401	3.2%	<.001	0.121	31	1.4%	31	1.4%	1	<0.001
Diabetes mellitus	523	23.2%	49,271	18.6%	<.001	0.113	523	23.2%	522	23.1%	.972	0.001
Atherosclerotic heart disease	434	19.2%	32,893	12.4%	<.001	0.188	434	19.2%	433	19.2%	.970	0.001
Obesity	1030	45.7%	92,894	35.0%	<.001	0.218	1030	45.7%	1041	46.2%	.742	0.010
Thyroid disorders	606	26.9%	43,211	16.3%	<.001	0.259	606	26.9%	411	18.2%	<.001	0.208
BMI	967	42.9%	78,670	29.7%	<.001	0.073	967	42.9%	968	42.9%	.976	0.001
Bariatric surgery procedures	33	1.5%	1869	0.7%	<.001	0.073	33	1.5%	27	1.2%	.436	0.023
Antiemetics	2174	96.4%	217,010	81.9%	<.001	0.480	2174	96.4%	1906	84.5%	<.001	0.413
Laxatives	1999	88.6%	157,242	59.3%	<.001	0.709	1999	88.6%	1455	64.5%	<.001	0.594

### 1 Year

Before propensity matching, there was a total of 323,049 patients: 2525 patients in the BTX usage with AWR cohort and 320,524 patients in the no BTX usage with AWR cohort.

After propensity matching, a total of 5016 patients were included in the analysis (Table 2). In the BTX usage with AWR cohort (*n* = 2508), the mean age was 55.8 (SD 16.3). There were 1449 (57.8%) females, 1028 (41.0%) males, and 31 (1.2%) unknown gender. The mean follow-up time was 295 days (SD 118.1). Medical history of the BTX usage with AWR cohort revealed 23.7% with diabetes mellitus, 19.5% with atherosclerotic heart disease, 26.6% with thyroid disorders, 45.3% classified as obese. In the no BTX usage with AWR cohort (*n* = 2508), the mean age was 55.8 (SD 16.3). There were 1449 (57.8%) females, 1028 (41.0%) males, and 31 (1.2%) unknown gender. The mean follow-up time was 289 days (SD 129.1). Medical history of the BTX usage with AWR cohort revealed 23.7% with diabetes mellitus, 19.5% with atherosclerotic heart disease, 17.2% with thyroid disorders, 44.7% classified as obese.

**Table 2.** Demographic and Operative Characteristics of Patients Undergoing AWR at 1 Year

	Before matching						After matching					
	BTX		No BTX				BTX		No BTX			
	2525		320,524				2508		2508			
	*n*/mean	%/SD	*n*/mean	%/SD	*P*-value	Standardized difference	*n*/mean	%/SD	*n*/mean	%/SD	*P*-value	Standardized difference
Age (years)	55.8	16.3	54.4	18.0	<.001	0.080	55.8	16.3	55.8	16.3	.999	<0.001
Gender												
Female	1449	57.8%	153,290	49.1%	<.001	0.174	1449	57.8%	1449	57.8%	1	<0.001
Male	1028	41.0%	150,240	48.2%	<.001	0.145	1028	41.0%	1028	41.0%	1	<0.001
Unknown	31	1.2%	8406	2.7%	<.001	0.145	31	1.2%	31	1.2%	1	<0.001
Diabetes mellitus	595	23.7%	55,845	17.9%	<.001	0.144	595	23.7%	595	23.7%	1	<0.001
Atherosclerotic heart disease	488	19.5%	39,076	12.5%	<.001	0.190	488	19.5%	488	19.5%	1	<0.001
Obesity	1135	45.3%	107,962	34.6%	<.001	0.219	1135	45.3%	1120	44.7%	.670	0.012
Thyroid disorders	666	26.6%	48,638	15.6%	<.001	0.271	666	26.6%	432	17.2%	<.001	0.227
BMI	1069	42.6%	92,610	29.7%	<.001	0.272	1069	42.6%	1069	42.6%	1	<0.001
Bariatric Surgery Procedures	36	1.4%	2123	0.7%	<.001	0.074	36	1.4%	19	0.8%	.021	0.065
Antiemetics	2415	96.3%	257,502	82.5%	<.001	0.458	2415	96.3%	2116	84.4%	<.001	0.412
Laxatives	2200	87.7%	181,690	58.2%	<.001	0.704	2200	87.7%	1570	62.6%	<.001	0.608

### 3 Years

Before propensity matching, there was a total of 299,206 patients: 2294 patients in the BTX usage with AWR cohort and 296,912 patients in the no BTX usage with AWR cohort.

After propensity matching, a total of 4558 patients were included in the analysis (Table 3). In the BTX usage with AWR cohort (*n* = 2279), the mean age was 55.5 (SD 16.9). There were 1293 (56.7%) females, 955 (41.9%) males, and 31 (1.4%) unknown gender. The mean follow-up time was 652 days (SD 415.6). Medical history of the BTX usage with AWR cohort revealed 23.4% with diabetes mellitus, 19.4% with atherosclerotic heart disease, 25.2% with thyroid disorders, 46.2% classified as obese. In the no BTX usage with AWR cohort (*n* = 2279), the mean age was 55.5 (SD 16.9). There were 1293 (56.7%) females, 955 (41.9%) males, and 31 (1.4%) unknown gender. The mean follow-up time was 662 days (SD 434). Medical history of the BTX usage with AWR cohort revealed 23.4% with diabetes mellitus, 19.4% with atherosclerotic heart disease, 18.8% with thyroid disorders, 44.1% classified as obese.

**Table 3.** Demographic and Operative Characteristics of Patients Undergoing AWR at 3 Years

	Before matching						After matching					
	BTX		No BTX				BTX		No BTX			
	2294		296,912				2279		2279			
	*n*/mean	%/SD	*n*/mean	%/SD	*P*-value	Standardized difference	*n*/mean	%/SD	*n*/mean	%/SD	*P*-value	Standardized difference
Age (years)	55.5	16.9	54.3	18.3	.003	0.064	55.5	16.9	55.5	16.9	.989	<0.001
Gender												
Female	1293	56.7%	141,097	48.5%	<.001	0.165	1293	56.7%	1293	56.7%	1	<0.001
Male	955	41.9%	141,363	48.6%	<.001	0.135	955	41.9%	955	41.9%	1	<0.001
Unknown	31	1.4%	8237	2.8%	<.001	0.103	31	1.4%	31	1.4%	1	<0.001
Diabetes mellitus	534	23.4%	51,398	17.7%	<.001	0.143	534	23.4%	534	23.4%	1	<0.001
Atherosclerotic heart disease	441	19.4%	36,576	12.6%	<.001	0.241	441	19.4%	441	19.4%	1	<0.001
Obesity	1054	46.2%	100,377	34.5%	<.001	0.241	1054	46.2%	1006	44.1%	.153	0.042
Thyroid disorders	574	25.2%	44,598	15.3%	<.001	0.247	574	25.2%	428	18.8%	<.001	0.155
BMI	979	43.0%	86,326	29.7%	<.001	0.278	979	43.0%	979	43.0%	1	<0.001
Bariatric surgery procedures	31	1.4%	2023	0.7%	<.001	0.066	31	1.4%	28	1.2%	.694	0.012
Antiemetics	2186	95.9%	241,264	83.0%	<.001	0.430	2186	95.9%	1965	86.2%	<.001	0.345
Laxatives	1989	87.3%	169,922	58.5%	<.001	0.685	1989	87.3%	1459	64.0%	<.001	0.563

### 5 Years

Before propensity matching, there was a total of 297,646 patients: 2449 patients in the BTX usage with AWR cohort and 295,197 patients in the no BTX usage with AWR cohort.

After propensity matching, a total of 4558 patients were included in the analysis (Table 4). In the BTX usage with AWR cohort (*n* = 2432), the mean age was 56.2 (SD 16.1). There were 1401 (57.6%) females, 1000 (41.1%) males, and 31 (1.3%) unknown gender. The mean follow-up time was 880 days (SD 670.0). Medical history of the BTX usage with AWR cohort revealed 23.7% with diabetes mellitus, 20.0% with atherosclerotic heart disease, 26.8% with thyroid disorders, 45.8% classified as obese. In the no BTX usage with AWR cohort (*n* = 2432), the mean age was 56.2 (SD 16.1). There were 1402 (57.6%) females, 999 (41.1%) males, and 31 (1.3%) unknown gender. The mean follow-up time was 955 days (SD 702). Medical history of the BTX usage with AWR cohort revealed 23.7% with diabetes mellitus, 19.9% with atherosclerotic heart disease, 18.5% with thyroid disorders, 46.3% classified as obese.

**Table 4.** Demographic and Operative Characteristics of Patients Undergoing AWR at 5 Years

	Before matching						After matching					
	BTX		No BTX				BTX		No BTX			
	2449		295,197				2432		2432			
	*n*/mean	%/SD	*n*/mean	%/SD	*P*-value	Standardized difference	*n*/mean	%/SD	*n*/mean	%/SD	*P*-value	Standardized difference
Age (years)	56.2	16.1	54.5	17.9	<.001	0.102	56.2	16.1	56.2	16.1	.969	0.001
Gender												
Female	1401	57.6%	140,746	49.0%	<.001	0.172	1401	57.6%	1402	57.6%	.977	0.001
Male	1000	41.1%	137,999	48.1%	<.001	0.141	1000	41.1%	999	41.1%	.977	0.001
Unknown	31	1.3%	8227	2.9%	<.001	0.112	31	1.3%	31	1.3%	1	<0.001
Diabetes mellitus	576	23.7%	51,741	18.0%	<.001	0.140	576	23.7%	576	23.7%	1	<0.001
Atherosclerotic heart disease	486	20.0%	36,790	12.8%	<.001	0.194	486	20.0%	485	19.9%	.971	0.001
Obesity	1115	45.8%	101,796	35.5%	<.001	0.212	1115	45.8%	1126	46.3%	.752	0.009
Thyroid disorders	652	26.8%	45,163	15.7%	<.001	0.273	652	26.8%	450	18.5%	<.001	0.199
BMI	1087	44.7%	91,593	31.9%	<.001	0.265	1087	44.7%	1087	44.7%	1	<0.001
Bariatric surgery procedures	36	1.5%	2027	0.7%	<.001	0.074	36	1.5%	27	1.1%	.254	0.033
Antiemetics	2346	96.5%	236,522	82.4%	<.001	0.469	2346	96.5%	2064	84.9%	<.001	0.407
Laxatives	2149	88.4%	169,356	59.0%	<.001	0.707	2149	88.4%	1572	64.6%	<.001	0.583

### Risk of Outcomes

At 6 months, BTX usage with AWR was associated with lower hernia recurrence (32.1% vs 35.6%; *P* = .013) but higher rates of infections (14.4% vs 10.5%; *P* < .001), wound disruption (8.7% vs 4.7%; *P* < .001), hematoma (1.3% vs 0.5%; *P* = .003), SSI (3.9% vs 3.4%; *P* = .002), and reoperation (10.7% vs 7.8%; *P* = .001). Seroma formation was clinically insignificant (Table 5).

**Table 5.** Comparative Outcomes of Patients Undergoing AWR With or Without BTA

Outcome	Follow-up	Botulinum AWR (%)	No Botulinum AWR (%)	*P*-value (difference)
All infections	6 months	14.40%	10.50%	<.001
	1 year	17.60%	11.90%	<.001
	3 years	22.60%	15.30%	<.001
	5 years	25.10%	16.80%	<.001
Wound disruption	6 months	8.70%	4.70%	<.001
	1 year	9.40%	5.50%	<.001
	3 years	10.80%	6.60%	<.001
	5 years	11.30%	5.00%	<.001
Seroma	6 months	2.10%	1.70%	.324
	1 year	2.20%	1.80%	.312
	3 years	2.60%	1.20%	.001
	5 years	2.80%	1.70%	.009
Hematoma	6 months	1.30%	0.50%	.003
	1 year	1.60%	0.90%	.031
	3 years	1.60%	0.70%	.002
	5 years	1.70%	1.00%	.047
SSI	6 months	3.90%	2.40%	.002
	1 year	4.60%	2.30%	<.001
	3 years	5.40%	3.30%	.001
	5 years	5.20%	2.40%	<.001
Reoperation	6 months	10.70%	7.80%	.001
	1 year	14.70%	9.10%	<.001
	3 years	17.60%	13.20%	<.001
	5 years	20.30%	13.40%	<.001
Hernia Recurrence	6 months	32.10%	35.60%	.013
	1 year	36.30%	37.70%	.32
	3 years	41.60%	43.00%	.323
	5 years	42.60%	44.80%	.112

This pattern persisted at 1, 3, and 5 years with differences in hernia recurrence becoming insignificant starting year 1 throughout year 5 (year 1: 36.3% vs 37.7%; *P* = .32; year 3: 41.6% vs 43.0%; *P* = .323; year 5: 42.6% vs 44.8%; *P* = .112). Additionally, the usage of BTX in AWR significantly increases seroma formation at year 3 (2.6% vs 1.2%; *P* = .001) and 5 (2.8% vs 1.7%; *P* = .009).

## DISCUSSION

In this multi-institutional, retrospective cohort study of greater than 5000 patients over 5 years, we found that preoperative BTX usage for AWR was associated with significantly reduced hernia recurrence rate but increased complication (infections, wound disruption, hematoma, SSI) and reoperation rates over time. These findings suggest that while BTX facilitates midline fascial closure in AWR procedures, the postoperative risks may outweigh the potential benefits long term.

Interestingly, these findings are partially consistent with previous reports and findings. Prior studies have concluded that preoperative use of BTX in hernia repair is a safe and effective practice that improves both short- and long-term results, specifically reduced hernia recurrence.^22,25,26^ The mechanism behind this stems from botulinum toxin temporarily blocking the release of acetylcholine at the nerve terminals, resulting in flaccid paralysis of the lateral abdominal muscles, causing the muscles to thin and lengthen, making fascial closure during surgical hernia repair more likely.^20,21,25,26^

However, these studies have also cited numerous limitations, such as small sample sizes, lack of standardization of BTX dosage/timing/technique, inconsistent follow up, and bias/conflicts that have made it difficult to link complication rates to BTX usage. As a result, the higher complication and reoperation rates seen in our study may be due to confounding by indication. BTX is often utilized in the repair of complex hernias, characterized by large width, multiple recurrences, loss of domain, and/or complex patient comorbidities, which intrinsically carry higher risk and a more challenging closure.^20,27,28^ There is a myriad of factors that contribute to surgical risk and a variety of ways that a hernia repair may be defined as complex. Therefore, the complex nature surrounding AWR itself may contribute to the high complication and recurrence rates seen in this study. Other possible explanations may include respiratory weakness and injection site hematomas caused by BTX itself. Case series conducted by Crowner et al^29^ and Zwaans et al^30^ reported that BTX can cause systemic muscle weakness and dysphagia, resulting in increased pulmonary complications. Furthermore, Ayoub^31^ reported in a case study a rare association between BTX injections and hematoma formation, potentially further complicating outcomes in AWR using BTX.

Nonetheless, the medical literature consistently reports that BTX-A injections for AWR are generally safe, with only minor complications observed. Large cohort studies and meta-analyses have not identified significant rates of systemic muscle weakness, dysphagia, or increased pulmonary complications attributable to BTX-A when used at standard dosages (typically 200-300 units of onabotulinumtoxinA or 500 units of abobotulinumtoxinA, injected bilaterally into the external and internal oblique muscles.^9,21,24,32,33^ Respiratory failure requiring intubation is rare and more commonly associated with the complexity of the hernia and the need for component separation, rather than BTX-A itself.^9^

These discrepancies can be attributed to the limited and low-quality evidence regarding the effectiveness of botulinum toxin A (BTA) in AWR, which is complicated by variability in study designs, surgical approaches, and methods of BTA application.^32^ Differences in technical protocols, treatment regimens, and patient selection criteria further underscore the urgent need for standardization. Additionally, as the use of BTA in this context is relatively recent, cross-study comparisons remain difficult, emphasizing the importance of uniform protocols.^21^ Well-designed randomized controlled trials with standardized BTA dosing, administration schedules, and follow-up assessments are essential to ascertain whether the reported associations with complications and outcomes are causal or merely associative.

Another important consideration is cost. Preoperative BTA requires relatively large doses compared with many aesthetic indications. Given commonly reported dosing (eg, 200-300 units of Botox^®^), medication alone may require 2-3 vials, and total cost can be substantial, particularly when including the procedural visit and image guidance.^21^ For context, manufacturer-reported wholesale acquisition cost list pricing and payer benchmarks suggest per-vial/per-unit costs that can translate into a 4-figure medication expense for typical AWR dosing ranges (exclusive of facility/professional fees). Although BTA is often justified by the potential to reduce the need for extensive surgical component separation and its downstream morbidity, robust cost-effectiveness data in AWR remain limited, and future work should evaluate whether any operative-time or morbidity reductions offset the added drug/procedural costs.^20^

### Aesthetic Implications and Relevance to Abdominoplasty

Although AWR is primarily functional, many patients undergoing AWR overlap with an aesthetic population (eg, post–massive weight loss, postpartum diastasis/ventral hernia, or patients considering/concurrently undergoing panniculectomy/abdominoplasty), where abdominal contour, scar quality, and soft tissue complications materially affect patient satisfaction. In this context, our finding that preoperative BTX was associated with higher rates of wound disruption, surgical site infection, hematoma/seroma at later time points, and reoperation has potential aesthetic ramifications because these events are strongly linked to widened scars, contour irregularity, delayed healing, and need for revisional procedures.

While BTX may transiently facilitate fascial medialization and early closure mechanics, the observed higher complication and reoperation profile over time could translate into an increased likelihood of unfavorable aesthetic sequelae (eg, 3hypertrophic/widened scars, lower abdominal scar migration, abdominal wall asymmetry, and recurrent bulge/contour distortion) and may complicate future body-contouring plans by increasing scar burden and soft-tissue compromise.

Accordingly, when BTX is considered in patients with concurrent or future abdominoplasty goals, surgeons should incorporate these potential aesthetic tradeoffs into shared decision-making and counseling, emphasizing meticulous patient selection and coordinated planning (eg, timing of BTX relative to any skin excision procedure, management of dead space/drains, and heightened vigilance for wound events). Future prospective studies should include patient-reported and aesthetic endpoints (eg, scar assessment, contour satisfaction, bulge recurrence, and revision rates) to better define whether BTX's mechanical benefits can be achieved without sacrificing cosmetic outcomes.

### Strengths and Limitations

There are many notable strengths to this study. First, this study includes a large sample size, the largest to date in this field, drawn from a global federated database, enhancing the generalizability of our findings. Furthermore, outcomes are followed up for an extended duration of 5 years, which helps to evaluate for late complications and improves the reliability of findings. Propensity matching was also performed in this study, improving comparability between treatment and control groups and reducing bias.

Some limitations should be noted. Despite statistical matching, there is potential for unmeasured confounding. Additionally, possible coding mistakes/errors and underreporting of complications may have occurred. Lastly, as this is a retrospective claims/EHR-network study, granular procedural variables (eg, exact formulation, dilution, total dose, units per injection, number/location of injection points, image-guidance modality, and timing relative to surgery) are not consistently available and could not be standardized across centers and could potentially impact complication rates.

Future studies should aim to evaluate BTX in AWR within prospective, randomized controlled trials, with standardized protocols for dose, injection technique, and timing. This would minimize confounding by indication and allow for more definitive conclusions regarding safety and efficacy.

The findings of this study provide important clinical implications for both surgeons and patients. Surgeons' knowledge of these findings may help expand AWR options and aid in careful patient selection based on proper risk stratification. Patients' awareness of these findings may result in careful counseling with providers to ensure proper awareness of risks and benefits associated with BTX usage in AWR.

## CONCLUSIONS

Our findings reveal that BTX can be a helpful adjunct in complex AWR but does not come without long-term complications. Given that AWR is a common procedure, with hundreds of thousands of cases performed annually in the United States, there is opportunity to modify risk and potential outcomes through adequate counseling and careful patient selection. Awareness and incorporation of the potential long-term complications of BTX usage in AWR in counseling protocols and surgical risk assessment tools may lead to improved patient selection and decreased postoperative morbidity and mortality. In addition to functional outcomes, our findings may have aesthetic implications for patients undergoing concomitant or future panniculectomy/abdominoplasty, as higher wound morbidity and reoperation can adversely affect scar quality and abdominal contour. These considerations should be incorporated into preoperative counseling and patient selection.
